# Do trial managers know when sites will fail to recruit? Preliminary results from the estimating site performance (ESP) study

**DOI:** 10.1186/1745-6215-16-S2-P182

**Published:** 2015-11-16

**Authors:** Kirsty Shearer, Hanne Bruhn, Anne Duncan, Seonaidh Cotton, Shaun Treweek

**Affiliations:** 1 University of Aberdeen, Aberdeen, UK

## 

A substantial investment of public money is made each year to fund large multicentre clinical trials. Around half will not recruit to target and require extensions, revised sample size targets or will be closed leaving the clinical question unanswered. For multicentre trials, a great amount of time, effort and cost is spent setting up clinical sites. One potential reason why a trial may fail to deliver is that some sites fail to fulfil their predicted recruitment potential. The ESP study explores whether there is a good (and simple) way of making informed decisions about which sites are worth investing scarce trial team resources.

ESP hypotheses that trial managers, who work closely with recruitment sites, will be able to predict whether a site will be a ‘good’ recruitment site before recruitment starts. If confirmed, such knowledge would enable resources to be targeted where they can have most benefit. In ESP, trial managers actively involved in setting-up sites during 2014/15 from one CTU's portfolio studies were asked to predict the recruitment success of each site they opened to recruitment. Predictions comprise a ‘Yes/No’ to whether the site would recruit to target, and why. Predictions are sealed in envelopes, collected, stored for 6 months and will be compared to actual recruitment in a meeting with the trial managers that made the predictions. We will present preliminary results based on over 20 predictions, which will indicate whether trial managers identify features of sites that accurately predict site recruitment performance.

